# Structural basis for inhibition of the voltage-gated sodium channel Na_V_1.7 by the tarantula toxin HWTX-I

**DOI:** 10.1016/j.jbc.2026.113130

**Published:** 2026-05-08

**Authors:** Hang Wu, Wenxing Wang, Zhaotun Hu, Yekui Yin, Dezheng Peng, Qing Zhou, Xi He, Bo Zhu, Guyu Zhong, Jie Zhong, Xinlong Hao, Yin Wang, Zhonghua Liu, Minzhi Chen, Jia Wang, Yunxiao Zhang, Xi Zhou

**Affiliations:** 1 The National and Local Joint Engineering Laboratory of Animal Peptide Drug Development, College of Life Sciences, Institute of Interdisciplinary Studies, Peptide and Small Molecule Drug R&D Platform, Furong Laboratory, Hunan Normal University, Changsha, China; 2 Key Laboratory of Research and Utilization of Ethnomedicinal Plant Resources of Hunan Province, College of Biological and Food Engineering, Huaihua University, Huaihua, China; 3 School of Chemistry and Chemical Engineering, Hunan Institute of Science and Technology, Yueyang, Hunan; 4 Research Department, Hunan Provincial People's Hospital and The First Afffliated Hospital of Hunan Normal University, Changsha, Hunan, China; 5 Department of Respiratory and Critical Care Medicine, Hunan Provincial People’s Hospital and The First Affiliated Hospital of Hunan Normal University, Changsha, Hunan, China

**Keywords:** HWTX-I, Na_V_1.7, pain, spider venom-derived peptide toxins, voltage-gated sodium channel, toxin-channel interaction

## Abstract

The voltage-gated sodium channel Na_V_1.7 is a pivotal therapeutic target for the developing novel, potent, and specific analgesics. HWTX-I, a peptide isolated from the venom of the tarantula *Ornithoctonus huwena*, is known to inhibit N-type calcium channels and has also been demonstrated to block tetrodotoxin-sensitive Na_V_ channels in dorsal root ganglion neurons. In this study, we show that HWTX-I potently inhibits Na_V_1.7 currents with an IC_50_ value of 40.6 ± 16.1 nM, but it does not significantly affect the voltage dependence of Na_V_1.7 steady-state activation and inactivation. Alanine-scanning mutagenesis revealed that residues K3, V5, F6, P11, N14, E15, W28, and K30 are critical for HWTX-I’s inhibitory activity against Na_V_1.7. Furthermore, site-directed mutagenesis analysis demonstrated that HWTX-I binds to the S3-S4 linker in domain II of Na_V_1.7, and the D816K mutation in Na_V_1.7 significantly abrogates the efficacy of the HWTX-I-Na_V_1.7 interaction. Molecular dynamics simulations combined with free energy decomposition analysis identified Na_V_1.7-D816 and HWTX-I-K3 as dominant energetic contributors to binding, consistent with a role for long-range electrostatic steering rather than a persistent short-range salt bridge. Collectively, these findings elucidate the structural basis of HWTX-I-Na_V_1.7 binding, identify the pharmacophore of the toxin, and provide valuable insights into the interactions between peptide toxins and Na_V_1.7. Therefore, this research may guide the future development of specific, safe, and efficacious Na_V_1.7 inhibitors for pain relief.

Pain is a distressing and unpleasant emotional experience associated with actual or potential tissue damage and is a serious public health problem (1). The primary source of pain signals perceived by humans stems from action potentials generated by voltage-gated sodium (Na_V_) channels and the subsequent propagation of electrical signaling. Among the nine Na_V_ channel subtypes (Na_V_1.1-Na_V_1.9), Na_V_1.7-Na_V_1.9 are mainly expressed in nociceptive sensory neurons, such as dorsal root ganglion (DRG) and trigeminal ganglia neurons. Notably, Na_V_1.7 plays an essential role in regulating cellular excitability by amplifying subthreshold stimuli for action potential generation (1, 2, 3, 4). Several pain-related disorders have been clinically associated with mutations in Na_V_1.7, including those caused by gain-of-function mutations, such as inherited erythromelalgia, small-fiber neuropathy, and paroxysmal extreme pain disorder (5, 6, 7, 8). Conversely, mutations in Na_V_1.7 that lead to a loss-of-function phenotype have been associated with congenital insensitivity to pain (9, 10, 11, 12). In a previous study, an epigenome engineering approach utilizing CRISPR-dCas and zinc finger proteins was employed to eliminate Na_V_1.7 expression *in situ* in mice, resulting in a long-lasting analgesic effect; in some cases, this effect persisted for up to 44 weeks (13). These genetic evidences provide compelling support for the hypothesis that inhibition of Na_V_1.7 represents a promising avenue for the development of analgesics.

A series of natural peptide toxins from venomous organisms have been identified as potent Na_V_1.7 modulators. These include KIIIA from cone snail (14), HNTX-III (15), ProTx-II (16), HWTX-IV (17), and GpTx1 (18) from tarantulas, along with μ-SLPTX-Ssm6a from centipedes (19). These molecules exhibit exceptional efficacy and subtype selectivity, and are considered potential lead molecules for analgesic development (20, 21). Spider-derived peptides, characterized by a high degree of structural complexity and the presence of multiple disulfide bonds (22), demonstrate enhanced selectivity through interactions with less conserved voltage-sensing regions. Currently, research on human (h)Na_V_1.7 analgesics is progressing steadily. This includes the discovery of novel inhibitors, structural optimization for improved pharmacokinetics, and structure and pharmacophore based drug design facilitated by recent breakthroughs in Na_V_1.7 structural elucidation (3, 18, 23, 24, 25, 26, 27, 28, 29).

HWTX-I was initially reported to inhibit the N-type Ca_V_2.2 channel (30). In this study, we have discovered that HWTX-I exhibits potent inhibitory activity against the Na_V_1.7 channel, displaying a specific subtype selectivity. Through comprehensive approaches combining alanine scanning mutagenesis, site-directed mutagenesis, and molecular dynamics (MD) simulations, we elucidated the molecular mechanism underlying the interaction between HWTX-I and Na_V_1.7. Our findings not only establish HWTX-I as a potential leader molecule for the development of Na_V_1.7-targeted analgesic drugs, but also provide structural- and pharmacophore-based insights to guide future therapeutic design.

## Results

### HWTX-I potently inhibits Na_V_1.7 channel

**Figure 1:**
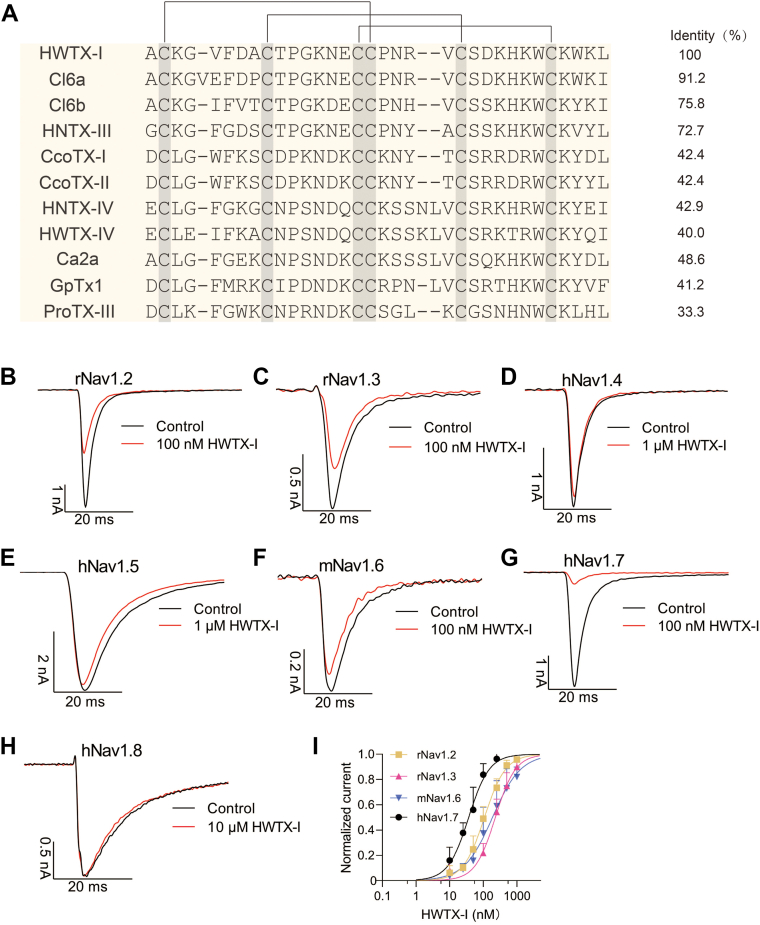
**HWTX-I belongs to the NaSpTx family 1 and is selective for Na_V_ subtybes (Na_V_1.2–1.8).***A*, sequence alignment of HWTX-I with other peptides derived from spiders using MAEG11.0. *B–H*, representative current traces of Na_V_1.2 (n = 5), Na_V_1.3 (n = 5), Na_V_1.4 (n = 5), Na_V_1.5 (n = 5), Na_V_1.6 (n = 5), Na_V_1.7 (n = 5), and Na_V_1.8 (n = 5) in the absence (*black*) and presence (*red*) of HWTX-1, respectively. *I*, concentration-dependent inhibitory curves show the effect of HWTX-I on Na_V_1.2 (n = 5), Na_V_1.3 (n = 5), Na_V_1.6 (n = 5), and Na_V_1.7 (n = 5). Data are presented as mean ± SD. Na_V_ channel, voltage-gated sodium channel.

HWTX-I, a neurotoxin isolated from the venom of the spider *Ornithoctonus huwena*, was previously established as a potent inhibitor of N-type Ca_V_ channels and later found to suppress tetrodotoxin-sensitive (TTX-S) Na_V_ channel currents in DRG neurons (30). Despite these findings, its activity against specific Na_V_ channel subtypes and the underlying mechanism of action remain unclear. Therefore, in this study, we aim to conduct an in-depth investigation of the effects of HWTX-I on various Na_V_ channel subtypes. A comparison of the amino acid sequences of HWTX-I with 11 additional peptide toxins derived from spider venom reveals intriguing similarities and potential functional implications (Fig. 1*A*). Structurally, these toxins, which are known to be potent inhibitors of the Na_V_1.7 channel, belong to NaSpTx family 1, the first family of spider venom toxins that target Na_V_ channels. Structurally, they are characterized by the presence of the classical inhibitor cystine knot motif and a conserved "WCK" motif located near the C terminus. The inhibitor cystine knot motif, a common structural feature in many venom peptides targeting ion channels, consists of a disulfide-bonded loop formed by cysteine residues (15, 31, 32). The conserved "WCK" motif may play a crucial role in the functional specificity of these toxins toward Na_V_1.7 channels. Given these structural similarities, it is reasonable to hypothesize that HWTX-I may similarly inhibit Na_V_1.7, potentially offering a new therapeutic option for pain treatment.

We investigated the effects of HWTX-I on HEK293T cells expressing Na_V_1.2, Na_V_1.3, Na_V_1.4, Na_V_1.5, and Na_V_1.7, as well as ND7/23 cells expressing Na_V_1.6 and Na_V_1.8, using whole-cell patch clamping. As shown in Figure 1, *B*–*H*, 100 nM HWTX-I completely inhibited the current amplitude for Na_V_1.7 and decreased the current amplitudes of Na_V_1.2, Na_V_1.3, and Na_V_1.6, but had no effect on the currents of Na_V_1.4, Na_V_1.5, and Na_V_1.8. Figure 1*I* shows that HWTX-I inhibited Na_V_1.7, Na_V_1.2, Na_V_1.3, and Na_V_1.6 in a concentration-dependent manner. The highest potency was observed against Na_V_1.7, with a half-maximal IC_50_ of 40.6 ± 16.1 nM. Progressively lower potency was observed against Na_V_1.2 (IC_50_ = 108.2 ± 33.5 nM, n = 5), Na_V_1.6 (IC_50_ = 187.8 ± 52.9 nM, n = 5), and Na_V_1.3 (IC_50_ = 228.7 ± 30.2 nM, n = 5) (Fig. 1*I*). In addition, 1 μM HWTX-I did not affect cardiac-expressed hERG (Kv11.1) channel (Fig. S1). Collectively, these data indicate that HWTX-I exhibits the highest potency against Na_V_1.7.

### Effect of HWTX-I on gating kinetics of Na_V_1.7 channels

**Figure 2:**
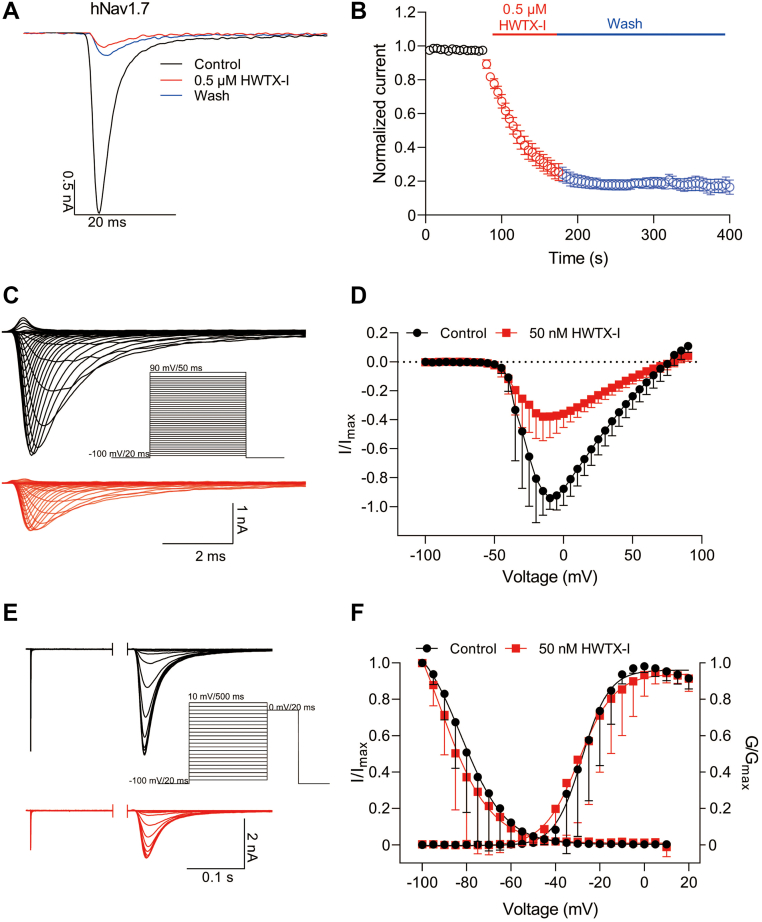
**HWTX-1 irreversibly inhibits Na_V_1.7 channel currents and gating kinetics affecting Na_V_1.7.***A*, representative current traces from Na_V_1.7 under control conditions (*black*) and with 0.5 μM HWTX-I (*red*). *Blue line* represent currents recorded 3 min after washing with bath solution. *B*, time course of the block development induced by 0.5 μM HWTX-I (n = 7) in peak currents of Na_V_1.7 channels, and recovery from inactivation after washing with bath solution. *C*, representative traces of current families were recorded from HEK293T cells expressing Na_V_1.7 in the absence or presence of 50 nM HWTX-I. The cells were held at −100 mV and stepped to potentials of −100 to +90 mV in 5-mV increments for 50-ms every 5-s. *D*, the current-voltage curves before and after treatment with 50 nM HWTX-1 at the Na_V_1.7 channel (n = 7). *E*, representative traces of voltage-dependent steady-state inactivation current families were recorded from HEK293T cells expressing Na_V_1.7 in the absence or presence of 50 nM HWTX-I. The noninactivated Na_V_1.7 currents were assessed by conducting a series of 500-ms prepulses ranging from holding potential −100 to 10 mV in 5-mV increments, followed by a 20-ms depolarization to 0 mV. *F*, curves of voltage-dependent steady-state activation (n = 7) and inactivation (n = 7) at the Na_V_1.7 channel before and after treatment with 50 nM HWTX-I. Data are presented as mean ± SD. Na_V_, voltage-gated sodium.

The voltage dependence of steady-state activation and inactivation is a crucial characteristic of Na_V_ channels (33). Numerous spider toxins function as gating modulators that regulate the voltage dependence of Na_V_ channel gating (31, 34). In this study, we demonstrate that HWTX-I potently inhibits Na_V_1.7. Consequently, our focus is on assessing the effects of HWTX-I on the activation and inactivation of Na_V_1.7 and elucidating its mechanism of action. Initially, we tested the time-dependent inhibition of Na_V_1.7 by 0.5 μM HWTX-I (Fig. 2, *A* and *B*). The results indicated that the toxin gradually inhibited Na_V_1.7 currents, with a time constant of 30 s for this inhibition. Notably, the inhibitory effect of HWTX-I remained unchanged even after washing with the external solution for up to 3 min, suggesting a potent and irreversible inhibitory effect. Subsequently, we investigated the effects of HWTX-I on the kinetics of activation and inactivation. Compared to the control, an inhibitory effect was observed at all test pulses after the addition of 50 nM HWTX-I (Fig. 2, *C* and *D*). The current-voltage (I-V) curves of Na_V_1.7 showed that HWTX-I did not alter the initial activation voltage or reversal potential of the Na_V_1.7 channel (Fig. 2*D*), implying that HWTX-I does not change the ion selectivity of the channel. In addition, we examined the effect of HWTX-I on the activation and inactivation of Na_V_1.7. To measure the steady-state inactivation curves, noninactivated Na_V_1.7 currents were elicited using a series of 500 ms prepulses ranging from −100 to 10 mV in 10 mV increments, followed by a 50-ms depolarization to 0 mV (Fig. 2*E*). As shown in Figure 2*F* and Table S1, HWTX-I exhibited no significant effects on the steady-state activation and inactivation kinetics of Na_V_1.7.

### Effects of HWTX-I mutants on Na_V_1.7 inhibition

**Figure 3:**
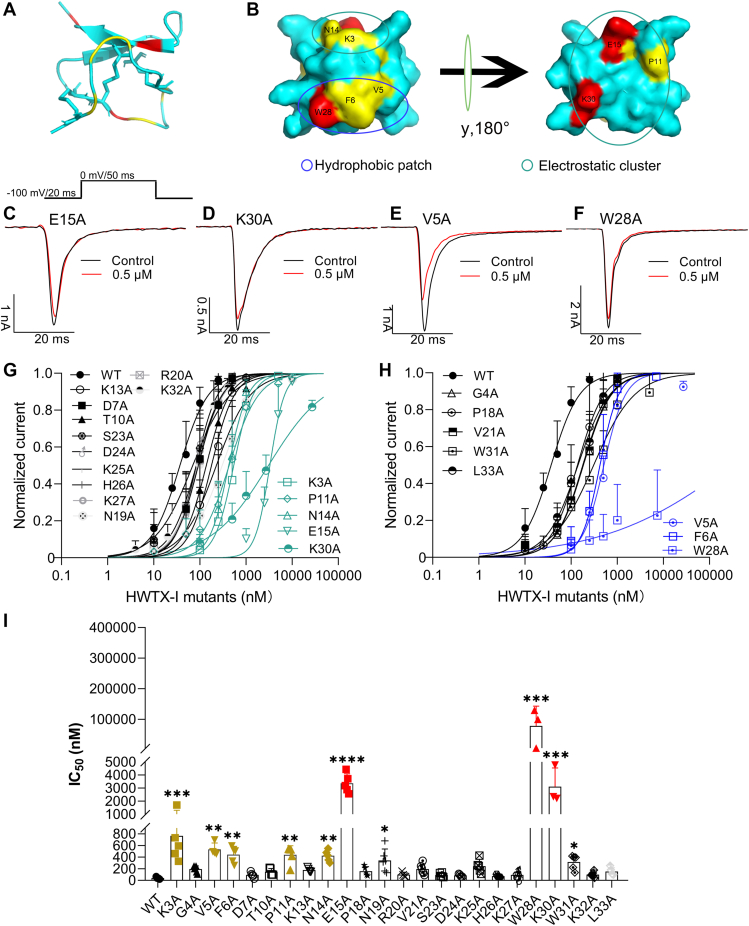
**Key amino acid residues of HWTX-I involved in Na_V_1.7 inhibition.***A* and *B*, three-dimensional spatial structure of HWTX-I (PDB: 1QK6). Richardson style diagrams of the backbone fold of HWTX-I (*A*). *B*, surface profiles of HWTX-I, plotted on the surface corresponding to the amino acid residues of Na_V_1.7 inhibitory activity. *C–F*, representative current traces from HEK293T cells expressing Na_V_1.7 in the absence (*black*) and presence (*red*) of HWTX-I mutants E15A (*C*, n = 5), K30A (D, n = 5), V5A (*E*, n = 5), and W28A (*F*, n = 5) at a concentration of 0.5 μM. The inset shows the voltage protocol. *G* and *H*, concentration-dependent inhibitory curves of HWTX-I mutants against Na_V_1.7 (n = 3–5). *I*, the bar diagram shows the IC_50_ values of the HWTX-I mutants on Na_V_1.7 (one-way ANOVA followed by Dunn's multiple comparisons test, n = 3–6). Data are presented as mean ± SD. ∗*p* < 0.05, ∗∗*p* < 0.01, ∗∗∗*p* < 0.001, ∗∗∗∗*p* < 0.0001 *versus* WT. Na_V_, voltage-gated sodium.

The molecular surface mapping of HWTX-I reveals a functionally amphipathic character, manifested by an asymmetric but nonplanar distribution of charged and hydrophobic residues across the peptide surface (Fig. 3, *A* and *B*). To delineate the structural determinants underlying its inhibitory activity, we performed alanine-scanning mutagenesis on all surface-exposed residues, excluding cysteines and native alanines. This systematic approach allowed us to evaluate the functional contribution of individual side chains within the disulfide-stabilized scaffold. Based on their chemical properties and functional impact, rather than strict geometric continuity, key residues were grouped into two major pharmacophore clusters: an electrostatic cluster and a hydrophobic patch.

Within the electrostatic cluster, alanine substitution of glutamate at position 15 (E15A) or lysine at position 30 (K30A) resulted in a pronounced loss of inhibitory potency. At 500 nM, both E15A and K30A exhibited markedly reduced inhibition of inward Na_V_1.7 currents (Fig. 3, *C* and *D*), corresponding to 82.9-fold and 76.6-fold decreases in activity, respectively (Table S2). Additional residues assigned to this cluster, including K3 (18.9-fold), N14 (10.4-fold), N19 (8.4-fold), and the structurally constrained P11 (10.9-fold), also contributed substantially to toxin–channel interactions. Importantly, the residues comprising this functional cluster are not contiguous on the protein surface; rather, they occupy spatially separated positions that cooperate to mediate interactions with the channel, primarily through ionic bonds and hydrogen bonding.

In parallel, a hydrophobic patch was identified as a critical anchoring element for channel binding. Substitution of tryptophan 28 (W28A) within this region resulted in a near-complete loss of activity (Fig. 3, *E* and *F*). Other hydrophobic residues, including V5, F6 and W31, exhibited 10.5-fold, 10.9-fold, and 6.5-fold reductions in inhibitory potency upon alanine substitution, respectively (Fig. 3, *G*–*I*). Furthermore, CD spectroscopy revealed that the mutants with markedly diminished inhibitory activity retained secondary structures largely similar to that of the WT peptide. This indicates that their reduced activity is not due to global structural disruption, but rather likely results from the mutation directly impairing specific interactions with Na_V_1.7 (Fig. S2). Collectively, these findings imply that the interaction between HWTX-I and Na_V_1.7 is influenced by both electrostatic forces and hydrophobic interactions.

### HWTX-I binds to the DII S3-S4 linker of Na_V_1.7

**Figure 4:**
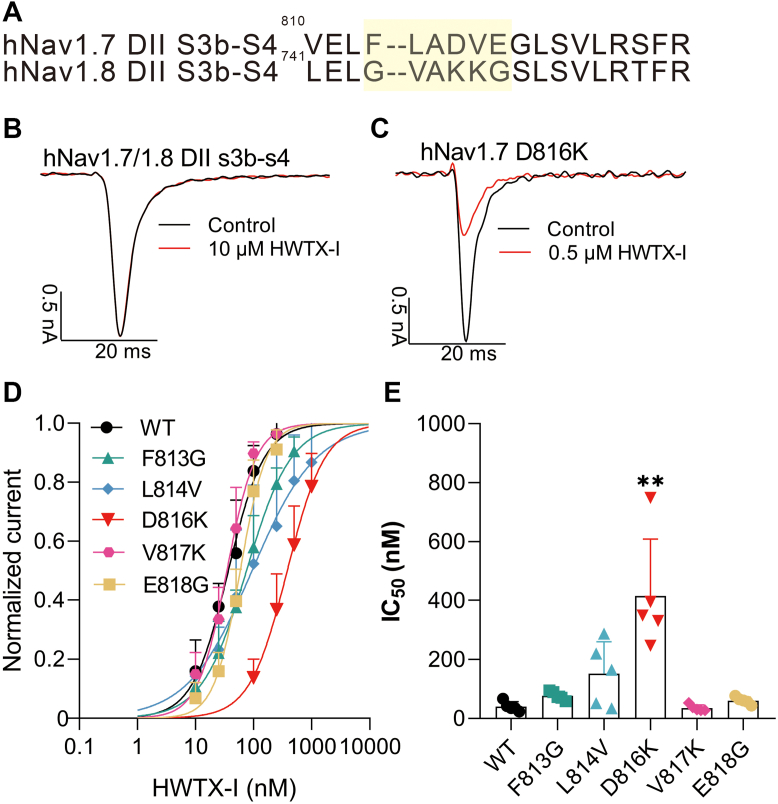
**The key amino acid residues of Na_V_1.7 DII S3-S4 linker involved in interaction with HWTX-I.***A*, sequence alignment corresponding to the DII S3-S4 region of the Na_V_1.7 and Na_V_1.8. The highlighted sequences show the regions swapped between Na_V_1.7 and Na_V_1.8. *B*, representative current traces from Na_V_1.7/1.8 DII S3-S4 chimera channels under control conditions (*black*) and with 10 μM HWTX-I. *C*, representative current traces from Na_V_1.7 D816K mutant channel under control conditions (*black*) and with 0.5 μM HWTX-I. *D*, concentration-dependent inhibitory curves show the effect of HWTX-I on WT and Na_V_1.7 mutant channels (n = 5). *E*, the bar graph presents HWTX-I's IC_50_ values for Na_V_1.7 mutant channels and the WT channel (one-way ANOVA followed by Dunn's multiple comparisons test, n = 5). Data are presented as mean ± SD. ∗∗*p* < 0.01 *versus* WT. Na_V_, voltage-gated sodium.

HWTX-I is a member of the NaSpTx1 family. These toxins are reported to act as neurotoxins site 4 (17, 21), which bind to the voltage sensor (S3-S4 linker) in domain II (DII) of Na_V_ channels to suppress channel opening. To investigate the binding site of HWTX-I on Na_V_1.7, we utilized the toxin’s selectivity for Na_V_ isoforms to construct a Na_V_1.7/Na_V_1.8 S3b-S4 chimeric channel (Fig. 4*A*). As shown in Figure 4*B*, 10 μM HWTX-I exerted almost no inhibitory effect on the chimeric channel, and the activity of HWTX-I was reduced by more than 300-fold compared to WT Na_V_1.7. To identify which amino acids in the Na_V_1.7 DII S3b-S4 region mediate HWTX-I binding, we designed a series of Na_V_1.7 channel mutants to map these key residues. As shown in Figure 4, *C* and *D*, residue D816 in Na_V_1.7 potently affected this interaction: 500 nM HWTX-I inhibited only ∼ 50% of the D816K mutant channel currents, whereas 100 nM HWTX-I almost completely inhibited WT Na_V_1.7 (Fig. 1*G*). Compared with WT Na_V_1.7, other Na_V_1.7 mutants exhibited varying degrees of reduced sensitivity to HWTX-I’s inhibitory activity (Fig. 4*E* and Table S3). These results suggest that D816 is a key amino acid residue governing the binding of HWTX-I to Na_V_1.7.

### Molecular docking and dynamics simulations of HWTX-I with Na_V_1.7

**Figure 5:**
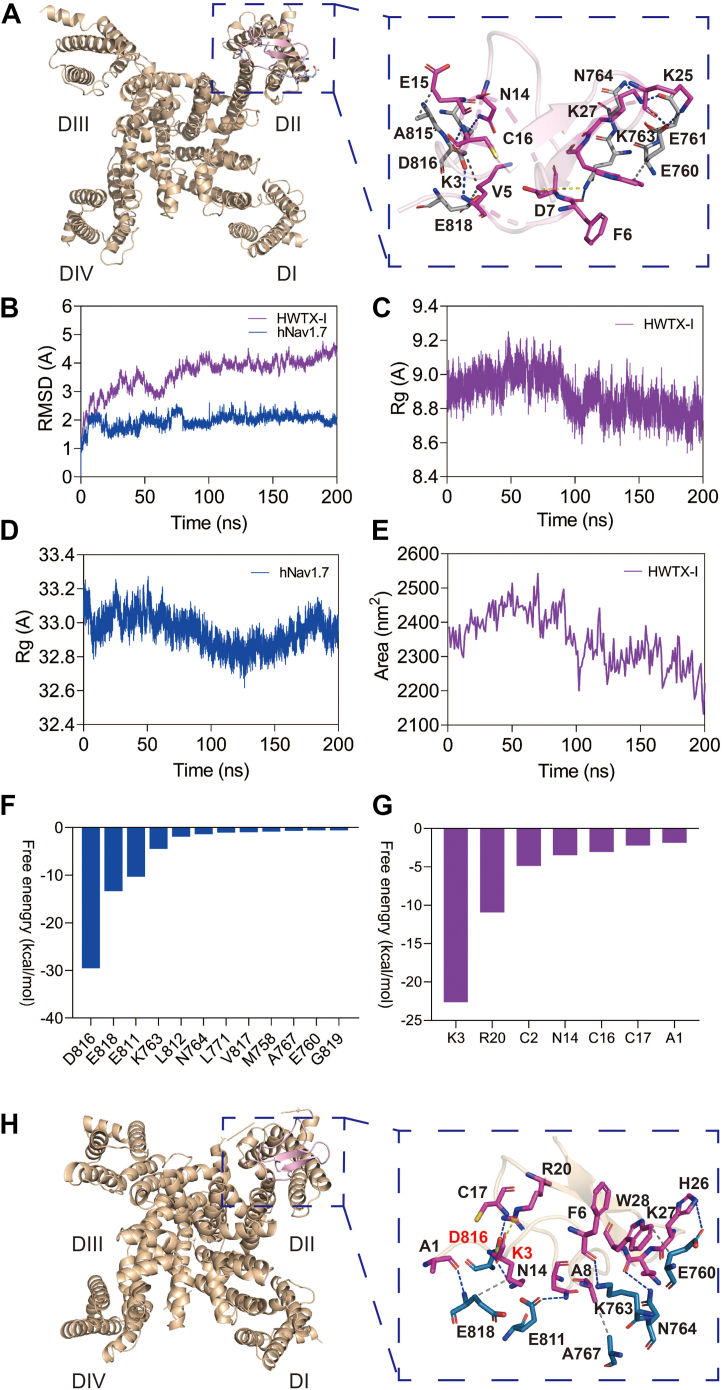
**Molecular docking and dynamics simulation of HWTX-I with Na_V_1.7.***A*, position of HWTX-I relative to the full-length α subunit of Na_V_1.7 channel and model of the HWTX-I/VSD-II complex, where ion channel residues and peptide residues are shown in *gray* and *purple*, respectively. *B–E*, the results of the molecular dynamics simulation of RMSD (*B*), Rg (*C* and *D*), and SASA (*E*) are shown, where *purple* represents the ion channel and *blue* represents the peptide. *F*, results of the channel residue energy decomposition of molecular dynamics. *G*, results of the peptide residue energy decomposition of molecular dynamics. *H*, results of the kinetic simulation display, where *purple* represents the peptide residues and *blue* is the protein. The D816-K3 interaction pair (*red*) represents the primary driving force underlying the binding between Na_V_1.7 and HWTX-I. Na_V_, voltage-gated sodium.

To explore the molecular basis of HWTX-I recognition by Na_V_1.7, an initial toxin–channel complex was first generated using molecular docking. This docking model was used to propose a plausible binding interface between HWTX-I and the voltage-sensing domain of Na_V_1.7, in which multiple charged and hydrophobic residues from both the toxin and the channel are positioned in close proximity (Fig. 5*A*). The docked structure suggests potential electrostatic complementarity between acidic residues on Na_V_1.7 and basic residues on HWTX-I, as well as possible hydrophobic contacts at the interface. However, given the static nature of docking and its tendency to overpredict residue contacts, this model was considered an initial structural hypothesis rather than definitive evidence of specific interactions. Therefore, unbiased MD simulations were subsequently performed to evaluate the stability of the complex and to refine the proposed interaction network under dynamic conditions.

In the docked model, several acidic residues on Na_V_1.7 (including E760, E761, and D816) were positioned near basic residues on HWTX-I (such as K3, K13, K25, and K30), consistent with favorable electrostatic complementarity. In addition, hydrophobic residues on the toxin (V5, F6, V21, W28, and W31) were oriented toward a hydrophobic region of the channel formed by residues including F813, L814, and V817. Polar residues, including the uncharged residue N14 and the acidic residue E15, were positioned to potentially contribute to interfacial polar and electrostatic interactions. In contrast, P11 is more plausibly interpreted to affect toxin activity indirectly by imposing local conformational constraints on the peptide backbone, consistent with its pronounced effect in alanine-scanning mutagenesis rather than direct interfacial contact.

Unbiased MD simulations were then conducted to evaluate the stability of the HWTX-I-Na_V_1.7 complex. Starting from the docked conformations (Fig. 5*A*), 200-ns of simulation were performed for the complex. The analysis of the RMSD was initially conducted for the 200-ns simulation, as illustrated in Figure 5*B*. The initial frame of each system was used as the reference structural coordinates to calculate the RMSD, reflecting the fundamental stability of the system throughout the simulation. The average RMSD of HWTX-I was 3.50 Å for the 200-ns simulations. The RMSD of HWTX-I exhibited a notable increase during the initial 50-ns, followed by a stability period around 100-ns, with fluctuations of less than 2 Å. Na_V_1.7 exhibited an average RMSD of 1.71 Å over the 200-ns simulation, stabilizing at approximately 25-ns. These findings indicate that the toxin-channel binding remains stable throughout the simulation.

The structural compactness of the HWTX-I was assessed before and after its binding to the Na_V_1.7 channel by calculating the Rg. During the 200-ns simulation, the Rg of HWTX-I exhibited a gradual decrease, indicating an increasingly compact structure (Fig. 5*C*). Meanwhile, the Rg of Na_V_1.7 decreased at approximately 50-ns, followed by a gradual increase in the Rg of the entire HWTX-I-Na_V_1.7 complex at 125-ns (Fig. 5*D*), although the overall Rg remained relatively low. These observations suggest enhanced structural ordering and compactness during HWTX-I binding, while Na_V_1.7 itself exhibits an overall structural contraction. Subsequently, we focused on the hydrophobic contact area between HWTX-I and Na_V_1.7 over the course of the simulated binding process. The findings showed that the contact area between HWTX-I and water progressively decreases as binding proceeds (Fig. 5*E*), a trend that correlates with the Rg trends observed earlier. This suggests that the hydrophobic region of HWTX-I is gradually encapsulated by Na_V_1.7, facilitating the formation of strong hydrophobic interactions between the toxin and the channel.

To further delineate residue-specific energetic contributions, free energy decomposition analysis was performed. This analysis identified Na_V_1.7 residue D816 and HWTX-I residue K3 as dominant contributors to the binding free energy (Fig. 5, *F* and *G*), in agreement with the pronounced reduction in inhibitory activity observed upon mutation of either site. Structural analysis of the representative MD cluster indicated that K3 is positioned near an acidic region of the channel, consistent with a role in long-range electrostatic steering rather than a persistent short-range salt bridge. In contrast, residues such as W28 are positioned to engage in stabilizing hydrophobic and aromatic-mediated interactions, providing a structural rationale for their strong functional effects. Residues including E15 and K30 likely contribute indirectly by influencing peptide orientation and binding microstates not fully captured by a single representative conformation (Fig. 5*H*). Collectively, this suggests support a hierarchical binding model in which a dominant electrostatic contribution identified by MD is complemented by additional residues that stabilize binding geometry and functional inhibition. The more compact channel conformation observed after MD simulation further suggests that binding of HWTX-I to the voltage-sensing domain of Na_V_1.7 may restrict conformational mobility, thereby impairing channel activation and reducing neuronal excitability.

## Discussion

The present study systematically investigated the inhibitory effects of HWTX-I, a neurotoxin isolated from the venom of *O*. *huwena*, on various Na_V_ channel subtypes and elucidated its molecular mechanism of action against Na_V_1.7, a key therapeutic target for pain management. Our findings demonstrate that HWTX-I exhibits high potency for Na_V_1.7, modulates Na_V_1.7 function through a potent irreversible mechanism without altering gating kinetics, identifies critical residues in HWTX-I that mediate channel inhibition, and confirms that HWTX-I binds to the DII S3-S4 linker of Na_V_1.7. These results not only expand our understanding of the functional diversity of spider venom toxins but also provide a theoretical basis for the development of HWTX-I as a potential novel analgesic drug.

To identify the structural determinants of HWTX-I’s inhibitory activity, we performed alanine-scanning mutagenesis on all surface-exposed residues. Our results revealed two major pharmacophore clusters: an electrostatic cluster and a hydrophobic patch. Three charged residues (K3, E15, and K30) were identified as critical residues, with their alanine substitution leading to 18.9-fold, 82.9-fold, and 76.6-fold decrease in activity, respectively. These results demonstrate that electrostatic interactions are critical for HWTX-I binding to Na_V_1.7. In the hydrophobic patch, W28 was found to be essential for activity, with W28A resulting in a near-complete loss of inhibition, while V5 and F6 also contributed to hydrophobic anchoring. These findings are consistent with the amphipathic nature of HWTX-I’s molecular surface and support a cooperative binding mechanism involving electrostatic steering by the polar cluster and hydrophobic anchoring by the hydrophobic patch. This dual pharmacophore model is similar to that of other NaSpTx1 toxins, such as HWTX-IV and HNTX-III (29, 35), which also relies on both electrostatic and hydrophobic interactions for channel binding, further confirming HWTX-I’s membership in this toxin family.

The DII VSD in the Na_V_1.7 channel serves as a critical interaction region for peptide toxins (36). Site-directed mutagenesis studies have further confirmed that the S3-S4 linker within DII is a pivotal binding segment for HWTX-I, and residue D816 has been identified as a key binding site. A comparison of the DII S3-S4 linker among the nine Na_V_ channel subtypes revealed that the position corresponding to D816 in Na_V_1.6 retains an aspartate residue. However, in Na_V_1.2, Na_V_1.3, and Na_V_1.4, this aspartate (D) is substituted by asparagine (N); in Na_V_1.5, it is substituted by arginine (R), an amino acid residue with the opposite charge. Consequently, we hypothesize that variations at the site corresponding to D816 underlie the subtype-specific binding of HWTX-I to Na_V_ channels.

The findings of this study have important implications for the development of novel analgesics. Na_V_1.7 is a validated pain target, and current Na_V_1.7 inhibitors are limited by poor subtype selectivity or off-target effects (36). HWTX-I’s high selectivity for Na_V_1.7 over the cardiac (Na_V_1.5) and skeletal muscle (Na_V_1.4) isoforms, coupled with its lack of effect on hERG channels, makes it a promising lead compound for analgesic development. The irreversible inhibition mechanism of HWTX-I also offers a potential advantage for long-term pain control, as it could reduce the frequency of drug administration. In addition, the identification of critical residues in HWTX-I (K3, E15, K30, and W28) and Na_V_1.7 (D816) provides a structural basis for rational drug design, allowing for the optimization of HWTX-I’s potency, selectivity, and pharmacokinetic properties. For example, modifying the pharmacophore clusters could enhance HWTX-I’s binding affinity or reduce its off-target activities, which is a common challenge for peptide-based drugs.

Collectively, our work elucidates the detailed molecular mechanism of HWTX-I's inhibition of Na_V_1.7. This mechanistic insight not only facilitates the rational engineering of Na_V_1.7-selective analgesic peptides based on HWTX-I, but also enhances the broader understanding of peptide toxin interactions with Na_V_ channels.

## Experimental procedures

### Peptide synthesis, oxidative folding, fraction purification, and concentration determination

The synthesis of HWTX-I and its mutants were performed using standard Fomc-based solid-phase peptide synthesis with the Fmoc (N-(9-fluorenyl) methoxycarbonyl)/tert-butyl strategy on Rink amide resin. The resulting peptides were C-terminally amidated, following our previously reported method (29, 37). Coupling reactions were facilitated by HOBt/TBTU/NMM, with the synthesis conducted at a 0.1 mmol scale. Following each elongation step, the terminal Fmoc group was removed using 20% (v/v) piperidine in N,N-dimethylformamide. Upon completion of the peptide chain assembly, the products were cleaved from the resin and simultaneously deprotected using a TFA-based cocktail (82.5% TFA, 5% phenol, 2.5% ethanedithiol, 5% thioanisole, and 5% deionized water) for 2 h at room temperature. The resulting crude peptides were precipitated in ice-cold diethyl ether, pelleted by centrifugation, washed, and lyophilized after dissolution in deionized water. Initial purification was achieved by semipreparative reverse-phase HPLC on a C18 column (10 × 250 mm, Welch Materials Inc), employing a linear gradient of 20 to 40% acetonitrile over 20 min at 3 ml/min. The collected linear peptides were then subjected to oxidative folding in a deoxygenated buffer (0.1 M NaCl, 0.05 M Tris-HCl, 20 mM guanidine hydrochloride, 5 mM GSH, 0.5 mM GSSG, pH 8.0) for 4 days at 25 °C. The folded products were isolated using the same HPLC conditions, and their identities were confirmed by MALDI-TOF mass spectrometry (AB SCIEX-TOF/TOF 5800). Only peptides exceeding 95% purity with the expected m/z values were retained. After lyophilization, the purified peptides were reconstituted in deionized water and stored as stock solutions. The efficiency of oxidative folding varied among mutants, likely due to positional effects of the introduced substitutions.

### Plasmid construction

Human (h)Na_V_1.5 and hNa_V_1.7, rat (r)Na_V_1.2, rNa_V_1.3, and mouse (m) Na_V_1.6 clones and beta subunit (β1 and β2-eGFP) clones were gifted by Dr Theodore R. Cummins (Indiana University School of Medicine). hNa_V_1.4 and hNa_V_1.8 clones were gifted by Prof. Yan Nieng (Tsinghua University). Mutations of Na_V_1.7 and Na_V_1.7/1.8 DII S3b-S4 chimaera were constructed using the Gene Tailor Site-Directed Mutagenesis system (Invitrogen), according to the manufacturer’s instructions.

### Cell culture and transfection

HEK293T and ND7/23 cells were cultured in Dulbecco’s modified Eagle’s medium supplemented with 10% fetal bovine serum, 2 mM L-glutamine, 100 U/ml penicillin, and 100 μg/ml streptomycin, and maintained at 37 °C with 5% CO_2_. For electrophysiological experiments, HEK293T cells were cotransfected with either (1) Kv11.1 (hERG), rNa_V_1.2, rNa_V_1.3, hNa_V_1.4, hNa_V_1.5, or mNa_V_1.6 channel plasmids along with enhanced green fluorescent protein (eGFP), or (2) hNa_V_1.7 (WT or mutant) channels coexpressed with β1 and β2-eGFP plasmids, while ND7/23 cells were transiently transfected with hNa_V_1.8 and eGFP using Lipofectamine 2000 (Invitrogen) according to the manufacturer’s instructions. Twenty-four hours post-transfection, fluorescent cells were selected for whole-cell patch-clamp recordings.

### Electrophysiological assays

We performed patch-clamp recordings on HEK293T or ND7/23 cells with an EPC 10 USB patch-clamp amplifier (HEKA Elektronik). The experimental protocol was adapted from our previously publication (38, 39). Cells were transferred to a custom perfusion chamber enabling rapid extracellular solution exchange. Pipettes with 2.0 to 2.5 MΩ resistance were fabricated from borosilicate glass capillaries *via* a two-step protocol using a PC-10 vertical puller (NARISHIGE). For Na_V_ channel experiments, the pipette solution contained 140 mM CsF, 10 mM NaCl, 1 mM EGTA, and 10 mM Hepes (pH 7.4 adjusted with CsOH), while the bath solution consisted of 140 mM NaCl, 2 mM CaCl_2_, 1 mM MgCl_2_, 5 mM KCl, 10 mM Hepes, and 10 mM glucose (pH 7.4 adjusted with NaOH). For Kv11.1 (hERG) recordings in HEK293T cells, the extracellular solution comprised 140 mM NaCl, 5 mM KCl, 2 mM CaCl_2_, 1 mM MgCl_2_, 10 mM Hepes, and 10 mM D-glucose (pH 7.3 adjusted with NaOH), whereas the pipette solution contained 140 mM KCl, 2.5 mM MgCl_2_, 10 mM Hepes, and 10 mM EGTA (pH 7.3 adjusted with KOH). TTX stock solutions (1 mM) were prepared in DMSO and added to the extracellular solution at 1 μM to block endogenous TTX-S Na^+^ currents during hNa_V_1.8 recordings in ND7/23 cells. All experiments were conducted at 20 to 25 °C using reagents from Sigma-Aldrich dissolved in ddH_2_O. Data were acquired with PatchMaster software, filtered at 5 kHz, and sampled at 20 kHz. To minimize voltage errors, 80 to 90% series resistance compensation was applied. Voltage-clamp recordings commenced 5 min after achieving whole-cell configuration to allow cytoplasmic equilibration with the pipette solution.

Conductance-voltage (G-V) relationships were determined by computing the conductance (G) at each test potential (V) using the equation G = I/(V − V_rev_), where V_rev_ denotes the cell-specific reversal potential. The resultant G-V data were then fitted to a Boltzmann equation: y = 1/(1 + exp [(V_1/2_ − V)/κ]), where V_1/2_ signifies the half-activation voltage, V represents the applied test potential, and κ corresponds to the slope factor. This approach enabled accurate quantification of conductance voltage-dependence, ensuring reproducibility across individual experimental recordings.

Steady-state inactivation curves were fitted to a Boltzmann equation: I/I_max_ = A + (1 - A)/{1 + exp [(V- V_1/2_)/κ],where V is the prepulse potential, V_1/2_ is the half-inactivation voltage, A represents the residual current fraction at saturating inactivation, and κ is the slope factor. This protocol allowed for systematic evaluation of the voltage dependence of channel availability, ensuring consistent quantification across experimental replicates.

Concentration-response relationships for HWTX-I and its analogs were analyzed using the Hill equation: y = 1 - 1/(1 + (x/IC50)n), x represents the concentration of HWTX-I or its analogs, and n is the Hill coefficient. During the experiment, cells transfected with Na_V_1.7 were held at −100 mV for 20 ms, with a stimulation voltage of 0 mV for 50 ms. The effect at each drug concentration was recorded as the current value, with stability maintained for more than two sweeps.

CDCD spectra were recorded for WT HWTX-I and its mutants using a Jasco J-1500 spectropolarimeter (Jasco). Measurements were performed at room temperature in a 0.01 M sodium phosphate buffer (pH 7.4) using a 1 mm path-length cuvette. Spectra were collected from 260 to 190 nm with a data interval of 0.1 nm, a scan speed of 100 nm/min, and an integration time of 2 s per data point. The observed ellipticity was converted to mean residue ellipticity, expressed in deg·cm^2^·dmol^−1^.

### Molecular docking

Using the membrane environment-specific energy function, the structure of the hNa_V_1.7-VSD2-NavAb channel (PDB: 6N4R) (40), and the NMR structure of HWTX-I (PDB: 1QK6) as templates (41), the haddock website (https://rascar.science.uu.nl/haddock2.4/) with membrane environment-specific energy function was employed to dock the HWTX-I model with the Na_V_1.7 model.

### MD simulation and calculation of free energy

MD simulations were conducted using the AMBER 20 software package (University of California). The system Na_V_1.7 was embedded into a pure palmitoyl-2-oleoyl-sn-glycero-3-phosphocholine membrane using Maestro. Hydrogen atoms for the proteins were added using the tleap module based on the ff19SB force field (42). The complexes were solvated in a TIP3P water box with a 12.0 Å buffer. Energy minimization of the complexes was performed using 2500 steps of steepest descent followed by 2500 steps of conjugate gradient. A 200 ps density equilibration was also carried out before the production run. Finally, 200 ns MD simulations were conducted for all systems under the NVT ensemble at 303 K, with a time step of 2 fs. Temperature was controlled using the Langevin thermostat, and pressure was controlled using the anisotropic Berendsen barostat. The RMSDs of all heavy atoms were determined with respect to the original conformation. After the completion of the MD simulations, the MD trajectories of each system were further analyzed using the cpptraj program. This analysis included RMSD, Rg, SASA. Binding free energies between the receptor protein and peptide were calculated by the Molecular Mechanics/Generalized Born Surface Area (MM/GBSA) method (43). Snapshots were taken every 200 ps from the last stable 200-ns MD trajectory and used to do the MM and GB calculations and obtained binding free energy.

### Data analysis

Data were analyzed by EPC 10 USB Patch Clamp Amplifier (HEKA, Elektronik), Igo Pro 6.10 A software (WaveMetrics), OriginPro 8 software (OriginLab Corporation), and Prism 7 software (GraphPad Software). Data were presented as mean ± SD, where n represented the number of separate experimental cells. Statistical analyses were performed using one-way ANOVA followed by Dunn's multiple comparisons test to compare the IC_50_ values of each mutant to the WT. Significant levels were set at *p* < 0.05 and the exact *p*-values are presented in Tables S2 and S3. Statistical analyses were performed with Prism 7 software.

## Data availability

All data will be shared upon requests to the corresponding author.

## Supporting information

This article contains supporting information.

## Conflict of interest

The authors declare that they have no conflicts of interest with the contents of this article
