# Supplementary material for: Structural basis for inhibition of the voltage-gated sodium channel NaV1.7 by the tarantula toxin HWTX-I
Source: J Biol Chem. 2026 May 8;302(6):113130. doi: 10.1016/j.jbc.2026.113130 (PMC13266018; doi:10.1016/j.jbc.2026.113130)
Supplement: Supplementary Figures [file mmc1.docx]

**Supplementary Figures and Figure legends**


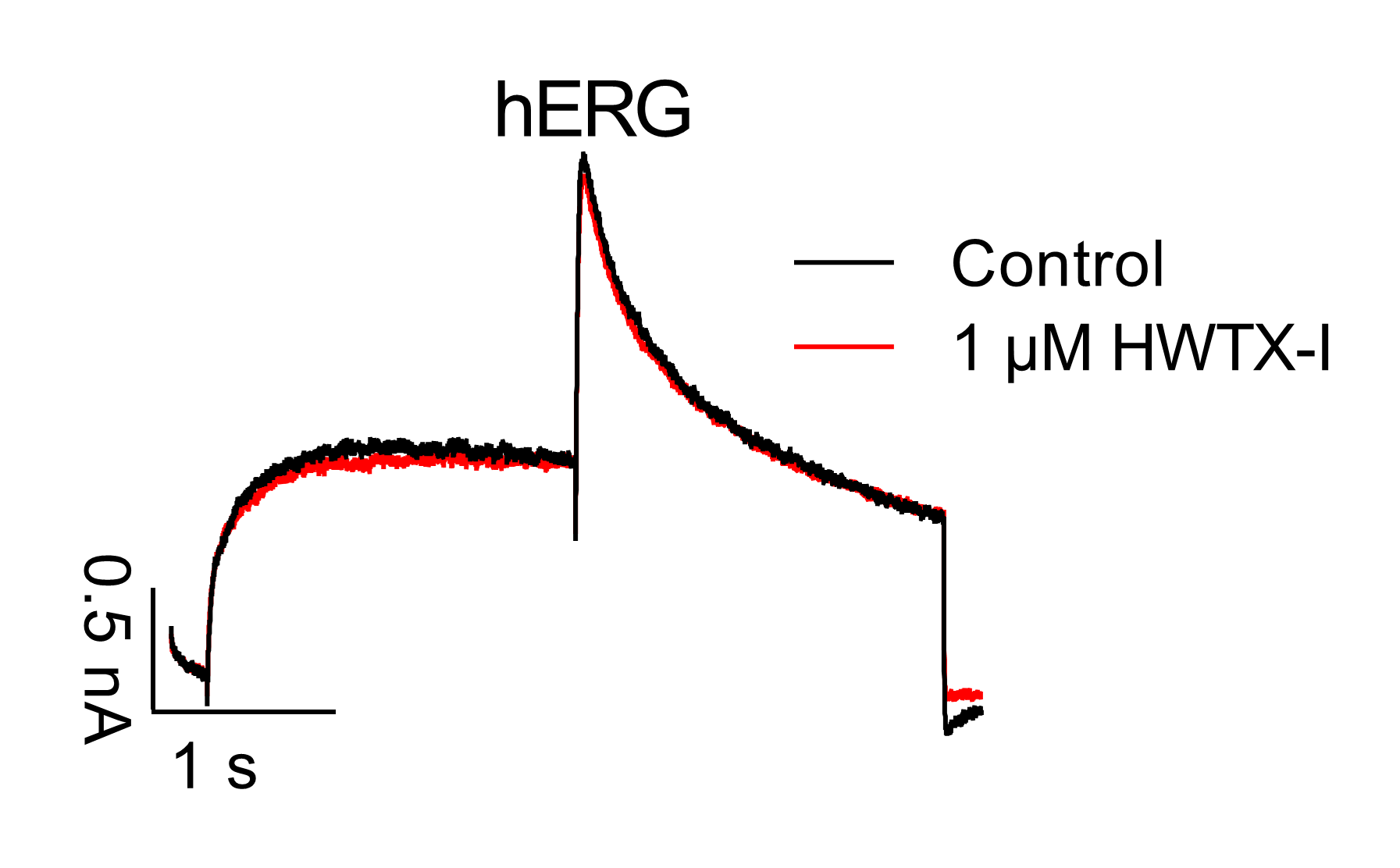


**Figure S1.** **Selectivity of HWTX-I for Kv11.1 (hERG).** Representative current alignments before (black) and after (red) the addition of HWTX-I, 1 μM HWTX-I had no effect on Kv11.1 (hERG) (n = 4).


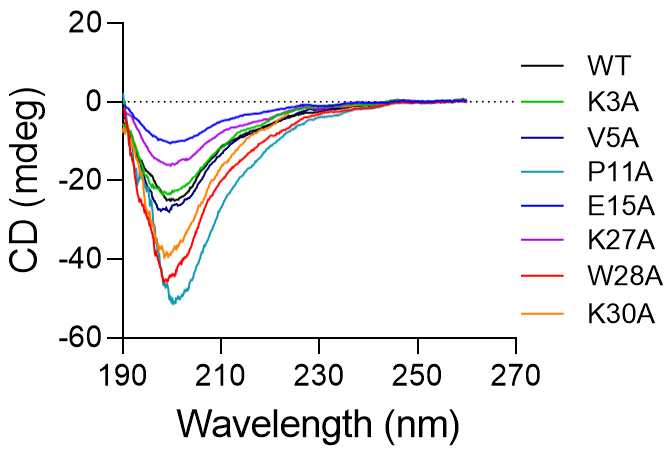


**Figure S2. CD spectra of WT and HWTX-I mutants.** Peptides were measured from 260 to 190 nm in 0.01 M sodium phosphate solution (pH 7.4) at room temperature.
