# Supplementary material for: Structural basis for inhibition of the voltage-gated sodium channel NaV1.7 by the tarantula toxin HWTX-I
Source: J Biol Chem. 2026 May 8;302(6):113130. doi: 10.1016/j.jbc.2026.113130 (PMC13266018; doi:10.1016/j.jbc.2026.113130)
Supplement: Supplementary — Tables [file mmc2.docx]

**Supplementary Tables**

**Table S1. The effects of 50 nM HWTX-I on voltage dependence of activation and inactivation of the Na_V_1.7 channel**

|  | Na_V_1.7 | | | | | |
| --- | --- | --- | --- | --- | --- | --- |
|  | **Voltage dependence of activation (mV)** | | | **Voltage dependence of inactivation (mV)** | | |
|  | V_1/2_ | κ | n | V_1/2_ | κ | n |
| **Control** | -28.0 ± 8.3 | 6.14 ± 1.4 | 7 | -82.3 ± 19.5 | 10.0±6.0 | 7 |
| **50 nM HWTX-I** | -29.4 ± 12.1 | 7.98 ± 1.7 | 7 | -92 ± 11 | 11.3±1.4 | 7 |

IC_50_ values are expressed as mean ± SD, where n represented the number of separate experimental cells.

| **Table S2. Statistical analysis of IC_50_ values for HWTX-I mutants compared to WT** | | | | |
| --- | --- | --- | --- | --- |
| Mutant | IC_50_ (nM) | n | p value vs WT | Significance |
| WT | 40.6 ± 16.1 | 5 | — | — |
| K3A | 766.1 ± 549.2 | 5 | 0.0004 | *** |
| G4A | 193.7 ± 55.2 | 6 | 0.1847 | ns |
| V5A | 532.1 ± 112.3 | 4 | 0.0016 | ** |
| F6A | 444.5 ± 142.1 | 5 | 0.0018 | ** |
| D7A | 94.1 ± 46.3 | 5 | >0.9999 | ns |
| T10A | 146.3 ± 59.9 | 5 | >0.9999 | ns |
| P11A | 441.9 ± 155.5 | 5 | 0.0025 | ** |
| K13A | 178.9 ± 35.2 | 5 | 0.3011 | ns |
| N14A | 424.0 ± 99.3 | 5 | 0.0021 | ** |
| E15A | 3367 ± 738 | 5 | <0.0001 | **** |
| P18A | 158.3 ± 78.7 | 5 | >0.9999 | ns |
| N19A | 343.0 ± 199.2 | 6 | 0.0144 | * |
| R20A | 98.6 ± 42.3 | 4 | >0.9999 | ns |
| V21A | 193.9 ± 91.9 | 6 | 0.275 | ns |
| S23A | 90.2 ± 34.6 | 6 | >0.9999 | ns |
| D24A | 87.1 ± 23.9 | 4 | >0.9999 | ns |
| K25A | 245.5 ± 119.0 | 5 | 0.0901 | ns |
| H26A | 71.9 ± 21.4 | 5 | >0.9999 | ns |
| K27A | 94.2 ± 51.0 | 6 | >0.9999 | ns |
| W28A | 78743 ± 64598 | 3 | 0.0002 | *** |
| K30A | 3113 ± 1438 | 3 | 0.0006 | *** |
| W31A | 316.0 ± 127.6 | 5 | 0.0289 | * |
| K32A | 99.9±42.8 | 6 | >0.9999 | ns |
| L33A | 152.0 ± 66.1 | 6 | >0.9999 | ns |
| IC_50_ values are expressed as mean ± SD. The one-way ANOVA followed by Dunn's multiple comparisons test, ^*^p < 0.05, ^**^p < 0.01, ^***^p < 0.001, ^****^p < 0.0001 versus WT. Where n represented the number of separate experimental cells. | | | | |

| **Table S3. Statistical analysis of IC_50_ values for Nav1.7 mutants compared to WT** | | | | |
| --- | --- | --- | --- | --- |
| Mutant | IC50 (nM) | n | p value vs WT | Significance |
| WT | 40.6 ± 16.1 | 5 | — | — |
| F813G | 77.3 ± 15.0 | 5 | 0.2621 | ns |
| L814V | 152.1 ± 108.5 | 5 | 0.3919 | ns |
| D816K | 414.2 ± 194.4 | 5 | 0.0019 | ** |
| V817K | 35.4 ± 10.5 | 5 | >0.9999 | ns |
| E818G | 60.8 ± 12.1 | 5 | >0.9999 | ns |
| IC_50_ values are expressed as mean ± SD. The one-way ANOVA followed by Dunn's multiple comparisons test, ^**^p < 0.01versus WT. Where n represented the number of separate experimental cells. | | | | |
